# The impact of hearing impairment and hearing aid use on progression to mild cognitive impairment in cognitively healthy adults: An observational cohort study

**DOI:** 10.1002/trc2.12248

**Published:** 2022-02-22

**Authors:** Magda Bucholc, Sarah Bauermeister, Daman Kaur, Paula L. McClean, Stephen Todd

**Affiliations:** ^1^ Cognitive Analytics Research Lab School of Computing Engineering & Intelligent Systems Ulster University Londonderry UK; ^2^ Department of Psychiatry University of Oxford Oxford UK; ^3^ Northern Ireland Centre for Stratified Medicine Biomedical Sciences Research Institute Ulster University Londonderry UK; ^4^ Altnagelvin Area Hospital Western Health and Social Care Trust Londonderry UK

**Keywords:** cognitive decline, cognitive impairment, dementia, dementia prevention, hearing aid, hearing impairment, hearing loss, mild cognitive impairment, preventive intervention

## Abstract

**Introduction:**

We assessed the association of self‐reported hearing impairment and hearing aid use with cognitive decline and progression to mild cognitive impairment (MCI).

**Methods:**

We used a large referral‐based cohort of 4358 participants obtained from the National Alzheimer's Coordinating Center. The standard covariate‐adjusted Cox proportional hazards model, the marginal structural Cox model with inverse probability weighting, standardized Kaplan‐Meier curves, and linear mixed‐effects models were applied to test the hypotheses.

**Results:**

Hearing impairment was associated with increased risk of MCI (standardized hazard ratio [HR] 2.58, 95% confidence interval [CI: 1.73 to 3.84], *P *= .004) and an accelerated rate of cognitive decline (*P < *.001). Hearing aid users were less likely to develop MCI than hearing‐impaired individuals who did not use a hearing aid (HR 0.47, 95% CI [0.29 to 0.74], *P* = .001). No difference in risk of MCI was observed between individuals with normal hearing and hearing‐impaired adults using hearing aids (HR 0.86, 95% CI [0.56 to 1.34], *P* = .51).

**Discussion:**

Use of hearing aids may help mitigate cognitive decline associated with hearing loss.

## 1 BACKGROUND

Mild cognitive impairment (MCI) describes a condition associated with demonstrable decline in cognitive abilities greater than normal age‐related changes but not severe enough to meet diagnostic criteria for dementia.^1^ MCI is common among older adults, with the prevalence estimates for those aged 65 or over ranging between 16% and 23%^1^ and the annual conversion rate to dementia and Alzheimer's disease (AD) of 9.6% and 8.1%, respectively.^2^

While numerous studies have investigated the impact of interventions aimed at preventing the onset of dementia in individuals with MCI, including exercise,^3^ antidepressant treatment^4^, non‐invasive brain stimulation,^5^ stress reduction,^6^ and the optimal control of vascular risk factors,^7^ there is a need for further research aimed at identifying prevention strategies earlier in the dementia process. The multidomain Finnish Geriatric Intervention Study to Prevent Cognitive Impairment and Disability (FINGER) intervention trial, combining lifestyle interventions, drug treatment, and cognitive training, demonstrated a beneficial effect of lifestyle intervention against cognitive decline among individuals with elevated risk of developing dementia.^8^ On the other hand, the findings of the Multidomain Alzheimer Preventive Trial (MAPT) and the Prevention of Dementia by Intensive Vascular Care (PreDIVA) randomized clinical trial (RCT) showed no significant impact of the multidomain lifestyle interventions on cognitive decline and incident all‐cause dementia, respectively.^9^, ^10^ Additional research is needed to ensure a robust evidence base and provide insight into the potential of different lifestyle interventions aimed at preventing cognitive decline and the hypothesized mechanisms of change underlying these interventions.

Hearing impairment is the third most common chronic health condition, with the prevalence of about 33% in individuals aged 65 years and older.^11^ Hearing loss and cognitive impairment often occur together, which creates the question of whether cognitive decline is caused by hearing loss and thus whether audiological rehabilitation through hearing aids or cochlear implants can reduce the risk of cognitive deterioration. The Lancet Commission on Dementia Prevention, Intervention, and Care estimated that failure to treat hearing impairment may account for up to 9% of dementia cases, assuming that there is in fact a causal relationship between auditory and cognitive decline.^12^ Several observational studies demonstrated that hearing impairment is associated with accelerated cognitive decline^13^, ^14^, ^15^, ^16^ and a higher risk of incident dementia.^12^, ^15^, ^17^ Furthermore, Lin et al.^17^ found a strong connection between the severity of hearing loss and dementia risk, with individuals suffering from mild to severe hearing loss having a 2‐ to 5‐fold increased risk of incident all‐cause dementia compared to those with normal hearing. The potential mechanistic hypotheses that could account for this association include the cognitive load theory; the changes in brain structure caused by the impoverished sensory input and decreased social interaction.^18^

Based on these reports, hearing rehabilitative strategies have been proposed as a way to restore deficits in cognitive function. The growing evidence from observational studies shows that hearing aid use is associated with better cognition, slower cognitive decline,^15^, ^19^, ^20^, ^21^ and reduced risk of developing all‐cause dementia.^19^ No significant effect of hearing aid use on cognition was found in some studies.^22^, ^23^ The discrepancies in the results across studies may arise from differences in the design, population size, selection bias, and methodological approaches used. To date, limited information on the effect of hearing treatment on cognitive function is available from RCTs. So far, the preliminary results from the Aging and Cognitive Health Evaluation in Elders (ACHIEVE) study demonstrated a significant effect of hearing aid use on composite memory scores after 6 months of follow‐up; however, improvement in other cognitive outcomes (language, executive function, global function) was not observed.^24^ Few RCTs further exploring the relationship between auditory impairment and cognition are ongoing.^25^, ^26^, ^27^

**HIGHLIGHTS**

- Hearing loss is associated with higher risk of mild cognitive impairment (MCI).
- Hearing loss is associated with accelerated cognitive decline.
- Hearing aid use is associated with lower risk of MCI and slower cognitive decline.
- People with normal hearing and hearing aid users have similar risk of MCI.
- Quality audiology screening might prove an effective dementia prevention strategy.

**RESEARCH IN CONTEXT**

1. **Systematic Review**: The authors reviewed the literature using PubMed. Although several studies evaluated the relationship between hearing impairment and cognitive decline, only two studies, with contradictory findings, have assessed the risk of developing mild cognitive impairment (MCI) in adults with and without hearing loss. The longitudinal studies investigating the association between hearing aid use and cognitive decline are scarce while no study has examined the role of hearing aids in healthy‐to‐MCI conversion.
2. **Interpretation**: Our findings show that hearing loss increases the risk of incident MCI while hearing aid use robustly reduces progression to MCI. Our findings highlight the importance of taking a proactive approach toward addressing hearing decline over time.
3. **Future Directions**: The article proposes a framework for the generation of new hypotheses. Examples include randomized clinical trials further exploring the relationship between auditory impairment and cognition and the hypothesized mechanisms that could account for this association.

Although there have been many studies that included cognitively normal adults as part of the study sample, when investigating the association between hearing loss and cognitive decline or dementia as an outcome, few studies have examined the impact of hearing impairment and hearing aid use on incident MCI.^28^, ^29^ MCI is important to consider as an earlier outcome on the trajectory of cognitive decline that could further improve our ability to identify which early detection measures, or combination of measures, obtained among individuals increase or decrease the likelihood of progression from normal cognition to the onset of clinical symptoms associated with cognitive impairment.

The aim of the present study was 3‐fold: (1) to examine the impact of hearing impairment on cognitive function and progression to MCI in cognitively healthy individuals; (2) to investigate the relationship among the use of hearing aids, incident MCI, and cognitive decline; and (3) to compare time to incident MCI and rate of cognitive decline between individuals with normal hearing and hearing‐impaired participants that used hearing aids.

## 2 METHODS

### 2.1 Study design and participants

**FIGURE 1:**
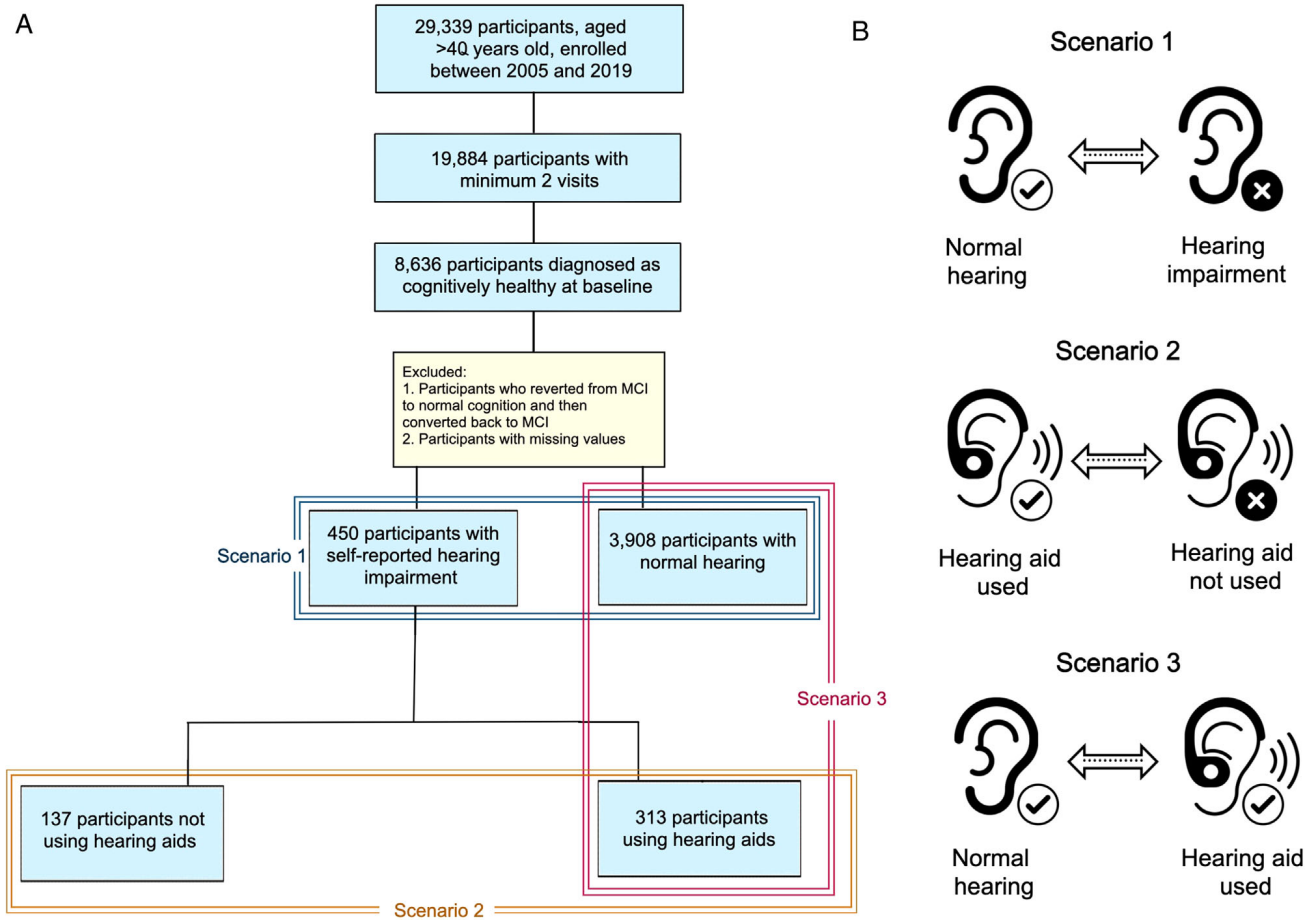
Flow diagram indicating (A) the selection of study participants and (B) schematic presentation of research scenarios considered in the study. Scenario 1 investigates the impact of hearing impairment on the progression from cognitively healthy to mild cognitive impairment (MCI). Scenario 2 examines the effect of hearing aid use on the progression from cognitively healthy to MCI. Scenario 3 compares the risk of incident MCI diagnosis in participants with normal hearing and hearing‐impaired subjects that used hearing aids. Data from 4358 participants, not diagnosed with cognitive impairment, 40 years of age or older, having more than one Alzheimer's Disease Research Center visit served as the initial sample for our study (Scenario 1). This included 450 subjects with hearing impairment and 3908 subjects without hearing impairment. Among 450 participants with hearing impairment, 313 were classified as hearing aid users and 137 as non‐users of hearing aids (Scenario 2). Information on hearing loss and hearing aid usage was collected via self‐report

The cohort we studied were volunteers followed up approximately annually at National Institute on Aging‐funded Alzheimer's Disease Research Centers (ADRCs). The National Alzheimer's Coordinating Center (NACC) maintains a database of standardized clinical and neuropathological information collected from these ADRCs.^30^ Consent was obtained from all participants at each ADRC under local institutional review oversight board. Data used in this study was the Uniform Data Set (UDS) including visits conducted between September 2005 and December 2018. The UDS data are collected using a standardized evaluation of participants enrolled in ADRC clinics and recorded directly on UDS forms (hard copy or electronic) during the evaluation process. Details about design, implementation, and rationale for the UDS have been published elsewhere.^31^ Data from individuals with normal cognition, 40 years of age or older at their first visit, with at least one follow‐up visit, served as the initial sample for our study. The records with incomplete clinical information were disregarded. Figure 1A shows a flowchart for the inclusion of participants and research scenarios considered in this study.

### 2.2 Procedures

MCI incidence was determined based on the clinical diagnosis made by a single clinician or through a consensus process. Diagnoses of MCI were established using the modified Petersen criteria.^32^ In addition, the longitudinally measured Clinical Dementia Rating Sum of Boxes (CDR‐SB) score was used to examine changes in cognitive performance in six domains: memory, orientation, judgment and problem solving, community affairs, home and hobbies, and personal care.^33^ The CDR‐SB was administered and scored by trained staff within their respective ADRCs.

Self‐reported hearing loss was evaluated through the response to the single question, “Without a hearing aid(s), is the subject's hearing functionally normal?” No information on the degree of hearing loss was provided in the NACC‐UDS. Although it was not reported to NACC whether participants with hearing impairment were wearing their hearing aids during cognitive testing, ADRCs were expected to use missing codes if it was indicated that hearing loss affected test performance. Records with missing codes were excluded from the analysis. Hearing aid use was also established based on self‐report. Hearing aid users were subsequently asked if the hearing aid provided them with “functionally normal hearing.” The positive response to this question implied that a participant did not demonstrate a reduced ability to carry out everyday activities, such as listening or talking, when wearing a hearing aid. All considered participants using hearing aids reported functionally normal hearing when wearing a device.

### 2.3 Statistical analysis

We used the two‐sided *t*‐test and Wilcoxon rank sum test to examine bivariate associations between predictor variables and outcomes for normally and non‐normally distributed continuous variables, respectively. The Shapiro‐Wilk test was used to determine whether the data significantly deviated from a normal distribution. Group comparisons for categorical measures were performed using the Chi‐square test.

Survival analysis was conducted to compare the time to the incidence of MCI between exposure groups for three primary outcomes (Figure 1B). First, we investigated the impact of hearing impairment on the progression from healthy to MCI (Scenario 1). Second, we measured the association between hearing aid use and progression from healthy to MCI (Scenario 2). Third, we examined the significance of the potential impact of hearing aid use by comparing the risk of incident MCI diagnosis in participants experiencing no hearing loss and hearing‐impaired subjects that used hearing aids (Scenario 3). Time to MCI conversion was measured from the baseline visit until the date when a patient was diagnosed with MCI or until the last registered visit for those who were lost to follow‐up (censored observations), with time measured in years since start of follow‐up. The censoring dates were defined as (1) the year of the NACC data freeze or (2) the year on which the patient's data were no longer available.

For the present analysis, we used two different survival models to evaluate the impact of hearing loss and hearing aid use on progression from cognitively healthy to MCI, namely, the standard Cox proportional hazards model (as benchmark), adjusted for baseline covariates, and the marginal structural Cox (MSC) model with inverse probability weighting (IPW).^34^ Herein, we refer to the standard covariate‐adjusted Cox proportional hazards model and the MSC model with IPW as the standard Cox model and IP‐weighted MSC model, respectively. IPW is a technique commonly used in survey sampling to adjust for sample selection bias and the potential confounding effects of both time‐varying and baseline covariates.^34^ As such, the method creates a so‐called pseudo‐population in which (1) the exposure is independent of the measured covariates and (2) drop‐out is not related to the measured exposure and covariates and thus, potential selection bias due to loss to follow‐up is accounted for. As a result, unlike the standard Cox model, the IP‐weighted MSC model allows for the estimation of the hazard ratio that can be interpreted, under certain assumptions (i.e., well‐defined exposures, the absence of unmeasured confounding, correct model specification, and positivity), in the same way as the hazard ratio is obtained in a RCT, in which no confounding and no loss to follow‐up are observed.^34^

We tested the proportionality of hazards assumption using the Schoenfeld residuals.^35^ The results suggested no meaningful departure from proportional hazards (*P* > .05). To account for events that occurred on the same date, we used Efron's approximation, which was previously shown to perform well in the presence of ties.^36^ The standard Cox model was adjusted for the baseline values of covariates including age, sex, years of education, diabetes, hypertension, hypercholesterolemia, years smoked, alcohol dependence, stroke, heart attack/cardiac arrest, body mass index (BMI), Geriatric Depression Scale (GDS) score, and hearing aid status (Scenario 1). BMI was categorized as underweight (<20.0 kg/m^2^), normal (≥20.0  and <27.5 kg/m^2^), or overweight (≥27.5 kg/m^2^), based on the suggestion that higher cut‐off points may be more appropriate for older adults.^37^ GDS scores were categorized as no depression (<5) and depression (≥5). For the IP‐weighted MSC model, we first calculated the exposure weights, based on the inverse of each participant's probability of the exposure, given the values of baseline (age at baseline, sex, years of education, and years smoked) and time‐varying covariates (hypertension, diabetes, hypercholesterolemia, alcohol dependence, stroke, heart attack/cardiac arrest, BMI, GDS score, and hearing aid status in Scenario 1).^38^ To test for potential non‐linear dependence in the data, we compared the model with only a linear term against the model with linear and cubic spline terms using a likelihood ratio test. The linearity assumption was satisfied for all continuous variables. Because some participants were lost to follow‐up throughout the study period, we also included censoring weights in the estimation process to account for selection bias due to loss to follow‐up. To increase statistical efficiency, the weights were stabilized. Next, the IP‐weighted MSC model was fitted by weighting participants according to their estimated exposure and censoring weights, with outcome time to incident MCI, and the hearing impairment or hearing aid status as the sole covariate. Using standardization by IP weights, we estimated standardized hazard ratios and Kaplan‐Meier (KM) survival curves. For all analyses, the reported *P*‐values were two‐sided and differences with *P* < .05 were considered statistically significant.

Additionally, to assess the impact of hearing impairment and hearing aid usage on cognitive function, we performed longitudinal analyses for Scenarios 1 to 3 using linear mixed effects models with individual CDR‐SB test scores as dependent variables (Models 1 to 3, respectively).^39^ Suitability for using a multilevel modeling approach was assessed by testing an “unconditional model” (“intercept only” model) in the first instance. Because the model showed significant between‐participant variation (*P* < .001), the use of multilevel modeling was supported. The effect of time, exposure allocation (i.e., hearing impairment or hearing aid status) were included as fixed effects. The inclusion of other covariates and time by exposure group interaction was judged against the model fit. Random intercepts and random slopes were modeled for each subject. We confirmed that all models, including the random intercept and the random slope, fitted the data significantly better than models incorporating only fixed effects (*P* < .001). The Akaike information criterion (AIC) was applied to evaluate the most plausible model in the set of models being tested.^40^ In all subsequent analyses, the significance of fixed and random effect parameters was evaluated using stepwise selection that started with the full model, then dropped predictor terms sequentially. Two‐tailed *P*‐values were obtained using Kenward‐Roger approximations for degrees of freedom.^40^

### 2.4 Sensitivity analysis

Sensitivity analysis for unmeasured confounding was performed to study the effect of potential unmeasured covariates on the standardized hazard ratios (HRs), in Scenarios 1 to 3, in IP‐weighted MSC models.^41^ We varied the assumed prevalence rates for the confounder among the exposed (10%, 15%, and 20% of the population) and unexposed groups (10%, 15%, and 20% of the population), and used three different values of HR (0.5, 1.5, and 2.0) for the association between the confounder and the outcome. This allowed us to assess how the inferences on the effects of exposures can be altered through an unknown variable under different simulations.

## 3 RESULTS

### 3.1 Participant characteristics

We analyzed a total of 4358 participants with no cognitive impairment at baseline. This included 450 (10.3%) subjects with hearing impairment and 3908 (89.7%) subjects without hearing impairment. Among 450 participants with hearing impairment, 313 were classified as hearing aid users while 137 were not using hearing aids. Out of 4358 participants, 416 converted to MCI during follow‐up. The mean follow‐up time was 4 years (standard deviation: 2.8, range: 2 to 12). The ages of the participants ranged from 40 to 80 years, with a median of 68 years (interquartile range: 63 to 73). The baseline characteristics of participants are shown in Table 1.

**TABLE 1:** Demographic characteristics of participants

	Scenario 1			Scenario 2			Scenario 3		
	Hearing impairment	Normal hearing	*P*‐value	Hearing aid used	Hearing aid not used	*P*‐value	Normal hearing	Hearing impairment, hearing aid used	*P*‐value
Sex, n (%), female	219 (48.7)	2775 (71.0)	<.001	149 (47.6)	70 (51.1)	.54	2775 (71.0)	149 (47.6)	<.001
Age, median (IQR)	73 (68‐77)	68 (63‐73)	<.001	73 (69‐77)	72 (67‐76)	.07	68 (63‐73)	73 (69‐77)	<.001
Education, mean (SD)^a^	16.2 (2.9)	15.8 (2.8)	.01	16.5 (2.7)	15.5 (3.3)	.002	15.8 (2.8)	16.5 (2.7)	<.001
CDR‐SB, mean (SD)	0.14 (0.46)	0.07 (0.30)	.001	0.13 (0.44)	0.17 (0.51)	.46	0.07 (0.30)	0.13 (0.44)	.02
BMI score, n (%)			.20			.89			.28
<20	13 (2.9)	127 (3.3)		10 (3.2)	3 (2.2)		127 (3.3)	10 (3.2)	
20–27	234 (52.0)	1853 (47.4)		163 (52.1)	71 (51.8)		1853 (47.4)	163 (52.1)	
≥27	203 (45.1)	1928 (49.3)		140 (44.7)	63 (46.0)		1928 (49.3)	140 (44.7)	
GDS score, n (%)			.24			.81			.43
<5	429 (95.3)	3768 (96.4)		299 (95.5)	130 (94.9)		3768 (96.4)	299 (95.5)	
≥5	21 (4.7)	140 (3.6)		14 (4.5)	7 (5.1)		140 (3.6)	14 (4.5)	
Diabetes, n (%)	52 (11.6)	475 (12.1)	.76	25 (8.0)	27 (19.7)	<.001	475 (12.1)	25 (8.0)	.03
Hypertension, n (%)	214 (47.6)	1810 (46.3)	.62	145 (46.3)	69 (50.4)	.47	1810 (46.3)	145 (46.3)	1
Hypercholesterolemia, n (%)	256 (56.9)	1913 (49.0)	.001	173 (55.3)	83 (60.6)	.30	1913 (49.0)	173 (55.3)	.03
Smoking, mean (SD)	8.9 (13.7)	9.0 (13.9)	.90	8.5 (13.1)	9.8 (15.2)	.38	9.0 (13.9)	8.5 (13.1)	.53
Alcohol dependence, n (%)	23 (5.1)	126 (3.2)	.05	16 (5.1)	7 (5.1)	1	126 (3.2)	16 (5.1)	.1
Stroke, n (%)	8 (1.8)	41 (1.1)	.16	7 (2.2)	1 (0.7)	.45	41 (1.1)	7 (2.2)	.09
Heart attack/cardiac arrest, n (%)	32 (7.1)	113 (2.9)	<.001	23 (7.3)	9 (6.6)	.84	113 (2.9)	23 (7.3)	<.001
Hearing aid use, n (%)	313 (69.6)	0 (0)	<.001	–	–		–	–	

Abbreviations: BMI, body mass index; CDR‐SB, Clinical Dementia Rarting Sum of Boxes; GDS, Geriatric Depression Scale; IQR, interquartile range; SD, standard deviation.

^a^ Measured as the number of years of education completed.

Given Scenario 1, participants with hearing impairment were significantly older (*P* <  .001), more educated (*P* =  .01), and had a higher mean CDR‐SB score (*P* =  .001) than individuals with normal hearing. A higher proportion of them were females (*P* <  .001) and suffered from hypercholesterolemia (*P* =  .001) and heart attack/cardiac arrest (*P* <  .001). In Scenario 2, non‐users of hearing aids had fewer years of education (*P* =  .002) and were more likely to suffer from diabetes (*P* < .001), compared to participants using hearing aids. In Scenario 3, participants with normal hearing were significantly younger (*P* <  .001), predominantly female (*P* <  .001), less educated (*P* <  .001), and had a lower mean CDR‐SB score (*P* =  .02) than hearing aid users. A lower proportion of them suffered from hypercholesterolemia (*P* =  .03) and heart attack/cardiac arrest (*P* <  .001), whereas the percentage of those with diabetes was higher (*P* =  .03).

### 3.2 Survival curves

**FIGURE 2:**
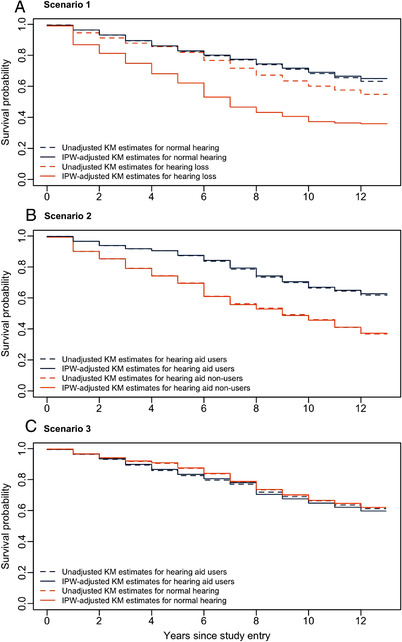
Unadjusted and inverse probability weighted Kaplan‐Meier (KM) survival curves showing cumulative mild cognitive impairment (MCI)‐free survival differences between: (A) participants with and without hearing loss (Scenario 1), (B) participants using and not using hearing aids (Scenario 2), (C) individuals with normal hearing and hearing‐impaired participants that used hearing aids (Scenario 3)

IP‐adjusted KM survival curves were constructed to compare the timing of incident MCI for different exposure conditions in Scenarios 1 to 3 (Figure 2). The comparison of these curves with the unadjusted KM estimates shows that although they have similar spread in Scenario 2 (Figure 2B), the large drop in the survival plot in Scenario 1 (Figure 2A) and a higher estimate of the survival probability for hearing aid users in Scenario 3 (Figure 2C) were driven by the implementation of IP weights. Because the median survival time was not reached at the time of report for some exposure groups in Scenarios 1 to 3, we used the restricted mean survival time (RMST) as an alternative measure of participants’ survival profile over time. We defined RMST as the average event‐free survival time up to the maximum possible follow‐up time of any participant within the design. As such, in Scenario 1, the estimated RMST (standard error [SE]) for individuals with normal hearing and those with hearing loss was 9.87 (SE: 0.06) and 7.29 (SE: 0.17; *P*  < .001), respectively. The 5‐year IP‐weighted rates of MCI‐free survival were 82.9% (95% confidence interval [CI], 81.7% to 84.1%) for participants with normal hearing and 62.2%, (95% CI, 53.6% to 72.1%) for participants with hearing loss. In Scenario 2, the RMST was 9.35 (SE: 0.16) for hearing aid users and 7.61 (SE: 0.31) for non‐users of hearing aids while IP‐weighted event‐free survival at 5 years was 87.5% (95% CI, 83.8% to 91.3%) and 69.4% (95% CI, 61.9% to 77.8%), respectively. In Scenario 3, the between‐group difference in RMST was 0.07 years and not statistically significant (*P* = .62).

### 3.3 Standard Cox model adjusted for baseline covariates

The standard Cox model adjusting for baseline covariates found that, compared to participants with normal hearing, those suffering from hearing loss were at significantly higher risk of incident MCI (HR 2.50, 95% CI, 1.72 to 3.63, *P* < .001; Scenario 1). A significantly reduced risk of MCI was associated with the use of hearing aids (HR 0.31, 95% CI, 0.19 to 0.53, *P* < .001; Scenario 2). No statistically significant differences in time to incident MCI diagnosis were found between hearing‐impaired individuals that used hearing aids and those with no hearing impairment (HR 0.85, 95% CI, 0.59 to 1.23, *P* = .4; Scenario 3). HR estimates for all considered covariates are summarized in Table 2.

**TABLE 2:** Unweighted and IPW HR and 95% CIs for the effect of hearing loss and hearing aid use on the progression from cognitively healthy to MCI

	Scenario 1		Scenario 2		Scenario 3	
	HR (95% CI)	*P*‐value	HR (95% CI)	*P*‐value	HR (95% CI)	*P*‐value
Standard Cox proportional hazards model adjusted for baseline covariates						
*Exposure of interest*						
Hearing impairment: No	Reference	–	–	–	–	
Hearing impairment: Yes	2.50 (1.72, 3.63)	<.001	–	–	–	–
Hearing aid use: No	–	–	Reference		–	–
Hearing aid use: Yes	–	–	0.31 (0.19, 0.53)	<0.001	–	–
Hearing impairment: No	–	–	–	–	Reference	
Hearing aid use: Yes	–	–	–	–	0.85 (0.59, 1.23)	0.4
*Covariates*						
Age, years	1.06 (1.05, 1.08)	<.001	1.08 (1.02, 1.13)	0.004	1.06 (1.05, 1.08)	<0.001
Education, years	0.96 (0.93, 0.99)	.03	0.98 (0.90, 1.06)	.58	0.96 (0.93, 0.99)	.03
CDR‐SB score	1.68 (1.48, 1.91)	<.001	1.88 (1.28, 2.75)	.001	1.66 (1.46, 1.90)	<.001
Sex: Female	0.73 (0.59, 0.90)	.003	1.08 (0.64, 1.84)	.77	0.71 (0.59, 1.23)	.002
BMI score						
20–27	Reference		Reference		Reference	
<20	1.47 (0.92, 2.35)	.1	1.34 (0.30,5.95)	.7	1.47 (0.91, 2.38)	.11
≥27	0.79 (0.64, 0.98)	.03	0.95 (0.56, 1.61)	.84	0.77 (0.61, 0.95)	.02
GDS score						
<5	Reference		Reference		Reference	
≥5	1.65 (1.05, 2.60)	.03	0.79 (0.23, 2.66)	.7	1.82 (1.14, 2.89)	.01
Diabetes, Yes	1.16 (0.88, 1.54)	.3	0.94 (0.46, 1.92)	.86	1.16 (0.86, 1.57)	.32
Hypertension, Yes	1.16 (0.94, 1.44)	.17	1.19 (0.68, 2.06)	.55	1.17 (0.93, 1.46)	.18
Hypercholesterolemia, Yes	1.04 (0.84, 1.27)	.74	1.05 (0.63, 1.77)	.85	1.06 (0.85, 1.31)	.62
Smoking, years	1.00 (0.99, 1.01)	.63	1.01 (0.99, 1.02)	.59	1.00 (0.99, 1.01)	.44
Alcohol dependence, Yes	1.32 (0.80, 2.19)	.28	0.33 (0.04, 2.58)	.3	1.40 (0.83, 2.35)	.21
Stroke, Yes	2.15 (1.20, 3.84)	.01	0.78 (0.10, 6.02)	.81	2.07 (1.13, 3.79)	.02
Heart attack/cardiac arrest, Yes	0.95 (0.60, 1.51)	.82	0.30 (0.07, 1.30)	.11	1.11 (0.69, 1.78)	.68
Hearing aid use, Yes	0.37 (0.23, 0.60)	<.001	–	–	–	–
Marginal structural Cox model with inverse probability (IP) weighting^a^						
*Exposure of interest*						
Hearing impairment: No	Reference		–	–	–	–
Hearing impairment: Yes	2.58 (1.73, 3.84)	<.001	–	–	–	–
Hearing aid use: No	–	–	Reference		–	–
Hearing aid use: Yes	–	–	0.47 (0.29, 0.74)	.001	–	–
Hearing impairment: No	–	–	–	–	Reference	
Hearing aid use: Yes	–	–	–	–	0.86 (0.56, 1.34)	.51

Abbreviations: 95% CI, 95% confidence interval; BMI, body mass index; CDR‐SB, Clinical Dementia Rating Sum of Boxes; GDS, Geriatric Depression Scale; HR, hazard ratio; IPW, inverse probability weighting; MCI, mild cognitive impairment.

^a^ IP weighted to account for confounding of exposure due to baseline covariates (age at baseline, sex, years of education, and years smoked), time‐varying covariates (hypertension, diabetes, hypercholesterolemia, alcohol dependence, stroke, heart attack/cardiac arrest, BMI, GDS score, hearing aid status [Scenario 1]), and selection bias due to drop out.

Scenario 1 investigates the impact of hearing impairment on the progression from cognitively healthy to MCI.

Scenario 2 examines the effect of hearing aid use on the progression from cognitively healthy to MCI.

Scenario 3 compares the risk of incident MCI diagnosis in participants with normal hearing and hearing‐impaired subjects that used hearing aids.

### 3.4 Inverse probability weighted marginal structural Cox model

All standardized HR estimates were similar to the HRs obtained from the standard Cox model (Table 2). After fitting the MSC model by weighting participants according to their estimated exposure and censoring weights, individuals with hearing loss were found to be at substantially higher risk of incident MCI (HR 2.58, 95% CI, 1.73 to 3.84, *P* < .001), compared to those with normal hearing (Scenario 1). In addition, the use of hearing aids was associated with a lower risk of healthy‐to‐MCI conversion (HR 0.47, 95% CI, 0.29 to 0.74, *P* = .001; Scenario 2). We found no statistically significant differences in risk of incident MCI between participants with normal hearing and hearing‐impaired adults that reported use of hearing aids (HR 0.86, 95% CI, 0.56 to 1.34, *P* = .51; Scenario 3).

### 3.5 Sensitivity analysis

Although IP‐weighted MSC models fully account for measured confounders and selection bias, they may still be susceptible to unmeasured confounding bias. Therefore, we conducted sensitivity analysis for unmeasured confounding to further assess the robustness of our results (Table 3). Sensitivity analysis for unmeasured confounding performed for Scenario 1 showed that hearing‐impaired individuals were at substantially higher risk of developing MCI compared to participants with normal hearing for all considered values of the strength of the confounder–outcome association, and the prevalence of potential confounder in the population. The results of sensitivity analyses obtained for Scenarios 2 and 3 were also consistent with those from the primary analysis; that is, the use of hearing aids was associated with lower risk of incident MCI in Scenario 2 and no difference in time to incident MCI was observed between patients with normal hearing and hearing‐impaired individuals using hearing aids in Scenario 3.

**TABLE 3:** Sensitivity analysis for unmeasured confounding in a marginal structural Cox model with IPW

		HR adjusted for unmeasured confounder (95% CI)^a^		
Prevalence of unmeasured confounder (%)		Unmeasured confounder HR 0.5	Unmeasured confounder HR 1.5	Unmeasured confounder HR 2.0
*Scenario 1*				
Normal hearing	Hearing impairment			
10	10	2.58 (1.73,3.84)	2.65 (1.78,3.94)	2.72 (1.83,4.05)
	15	2.51 (1.69,3.74)	2.58 (1.73,3.84)	2.65 (1.78,3.95)
	20	2.44 (1.64,3.64)	2.51 (1.69,3.74)	2.58 (1.73,3.84)
15	10	2.58 (1.73,3.84)	2.52 (1.69,3.75)	2.46 (1.65,3.67)
	15	2.64 (1.77,3.93)	2.58 (1.73,3.84)	2.52 (1.69,3.75)
	20	2.70 (1.81,4.02)	2.64 (1.77,3.93)	2.58 (1.73,3.84)
20	10	2.58 (1.73,3.84)	2.47 (1.66,3.67)	2.36 (1.59,3.52)
	15	2.70 (1.81,4.01)	2.58 (1.73,3.84)	2.47 (1.66,3.68)
	20	2.81 (1.89,4.19)	2.69 (1.81,4.01)	2.58 (1.73,3.84)
*Scenario 2*				
Hearing aid used	Hearing aid not used			
10	10	0.47 (0.29,0.74)	0.48 (0.30,0.76)	0.49 (0.31,0.78)
	15	0.45 (0.28,0.72)	0.47 (0.29,0.74)	0.48 (0.30,0.76)
	20	0.44 (0.28,0.70)	0.45 (0.28,0.72)	0.47 (0.29,0.74)
15	10	0.47 (0.29,0.74)	0.46 (0.29,0.73)	0.44 (0.28,0.71)
	15	0.48 (0.30,0.76)	0.47 (0.29,0.74)	0.46 (0.29,0.73)
	20	0.49 (0.31,0.78)	0.48 (0.30,0.76)	0.47 (0.29,0.74)
20	10	0.47 (0.29,0.74)	0.45 (0.28,0.71)	0.43 (0.27,0.68)
	15	0.49 (0.31,0.78)	0.47 (0.29,0.74)	0.45 (0.28,0.71)
	20	0.51 (0.32,0.81)	0.49 (0.31,0.78)	0.47 (0.29,0.74)
*Scenario 3*				
Normal hearing	Hearing aid used			
10	10	0.86 (0.56,1.34)	0.89 (0.57,1.37)	0.91 (0.59,1.41)
	15	0.84 (0.54,1.30)	0.86 (0.56,1.34)	0.89 (0.57,1.37)
	20	0.82 (0.53,1.27)	0.84 (0.54,1.30)	0.86 (0.56,1.34)
15	10	0.86 (0.56,1.34)	0.84 (0.55,1.31)	0.82 (0.53,1.28)
	15	0.88 (0.57,1.37)	0.86 (0.56,1.34)	0.84 (0.55,1.31)
	20	0.90 (0.58,1.40)	0.88 (0.57,1.37)	0.86 (0.56,1.34)
20	10	0.86 (0.56,1.34)	0.83 (0.53,1.28)	0.79 (0.51,1.23)
	15	0.90 (0.58,1.40)	0.86 (0.56,1.34)	0.83 (0.53,1.28)
	20	0.94 (0.61,1.46)	0.90 (0.58,1.39)	0.86 (0.56,1.34)

Abbreviations: CI, confidence interval; HR, hazard ratio; IPW, inverse probability weighting.

*Notes*: The selected prevalence rates for the unmeasured confounder among the exposed group were 10%, 15%, and 20% of the population. Three different values of HR, namely 0.5, 1.5, and 2.0, for the association between the confounder and the outcome were used. The prevalence of the unmeasured confounder in the unexposed group was varied from 10% to 20% to determine the extent to which its distribution under these conditions would need to be imbalanced to influence the statistical significance of the primary analysis. All models accounted for fixed‐time covariates (age at baseline, sex, years of education, and years smoked), time‐varying covariates (diagnosis of hypertension, diabetes, and hypercholesterolemia; alcohol dependence; stroke; heart attack/cardiac arrest; body mass index; Geriatric Depression Scale score; and hearing aid status [in Scenario 1]) and selection bias due to loss to follow‐up.

### 3.6 Longitudinal changes in cognitive function

In a complementary analysis, we assessed the impact of hearing impairment and hearing aid usage on cognitive function in Scenarios 1 to 3 using linear mixed effects models (Model 1 to 3, respectively) with CDR‐SB scores as dependent variables. In Model 1, for every 1‐year increase, CDR‐SB increased by 0.01 (*P < *.001). On average, individuals with hearing impairment tended to have 0.1 points higher CDR‐SB score compared to those without hearing loss (*P < *.001). Participants with normal hearing also showed less time‐related decline than individuals with hearing loss (*P = *.004). Accordingly, the annual rate of change in CDR‐SB was 0.012 points higher for individuals with hearing loss. Within Model 2, we found a significant effect of hearing aid status on the CDR‐SB score (*P = *.01). The CDR‐SB score reported for hearing‐impaired participants using hearing aids was, on average, 0.07 points lower than for non‐users of hearing aids. The mean annual rate of change in CDR‐SB for users and non‐users of hearing aids was 0.04 and 0.08 points, respectively. In Model 3, we observed a significant effect of follow‐up time on the CDR‐SB score (*P < *.001), with the annual rate of change in CDR‐SB of 0.006 points. Temporal changes in the CDR‐SB score for individuals without hearing loss and hearing‐impaired adults using hearing aids were statistically insignificant (*P = *.16).

## 4 DISCUSSION

Our study reveals that hearing impairment is independently associated with accelerated cognitive decline and higher risk of incident MCI. Hearing aid use is linked to lower rates of cognitive decline and reduced risk of incident MCI, with hearing aid users having more than 50% lower risk of MCI, compared to those not using hearing aids. Importantly, we demonstrated that no significant differences in risk of developing MCI and cognitive decline exist between participants experiencing no hearing loss and those diagnosed with hearing impairment using hearing aids. This implies that use of hearing aids may help mitigate cognitive decline associated with hearing loss, offering an actionable strategy to reduce the incidence of MCI. Sensitivity analyses did not affect the results of our primary analysis; hearing loss increases the risk of incident MCI while hearing aid use robustly reduces progression to MCI. The exposure effect of hearing loss and the treatment effect of hearing aids on incident MCI reported using the standard Cox model, persisted after adjusting for time‐dependent and time‐independent factors, and selection bias due to loss to follow‐up in MSC model with IPW and after accounting for unmeasured confounding.

Our results are consistent with several observational studies that showed a significant association between hearing loss and accelerated cognitive decline.^13^, ^14^, ^15^, ^16^ Although Heywood et al.^28^ reported that hearing loss was not associated with significantly higher risk of developing MCI, their model was not adjusted for hearing aid use. Given that use of hearing aids may help mitigate cognitive decline, hearing aid usage should be incorporated as a confounding factor when analyzing changes in cognitive function. Alternatively, the impact of hearing loss on cognition should be assessed by comparing participants with normal hearing and participants with hearing loss not using hearing aids. As such, Amieva et al.^15^ found a significant difference in the rate of change in cognitive scores between hearing‐impaired individuals not using hearing aids and controls while indicating no difference in cognitive decline between subjects with hearing loss using hearing aids and controls.

The potential beneficial treatment effect of hearing aids on cognitive decline observed in our study is consistent with conclusions of previous work.^19^, ^21^, ^42^, ^43^ In a prospective interventional study involving 34 elderly participants with hearing loss, cognitive function was significantly improved after 3 months of hearing aid use.^42^ Sarant et al.^43^ showed that treatment of hearing loss led to improvements in cognition and self‐reported listening disability after 18 months of hearing aid use, with >97% of participants reporting significant improvements in executive function. Hearing aid use was also associated with lower risk of incident dementia in individuals with MCI.^19^ The percentage of participants who had not developed dementia 5 years after the baseline MCI diagnosis was shown to be significantly higher for users (33%) than non‐users of hearing aids (19%).^19^

The interpretation of results of our study should be made in light of several limitations. First, the categorization of participants relied solely on self‐reported measures of hearing loss and hearing aid use. Although this may not be a limitation, as indicated in Oosterloo et al.,^44^ discrepancies between self‐reported hearing and audiometric assessment have been observed in other previous work.^45^ Further reported difficulties with using self‐report to estimate prevalence of hearing loss include people with similar hearing deficits reporting less disability and handicap as their age increases.^46^ Due to unavailability of data, we were unable to examine the impact of the degree of hearing impairment and measurement of how often hearing aids were used, or whether they were appropriately fitted, on the observed associations. In addition, given that hearing aid users wait an average of 7 to 10 years to seek help for hearing loss,^47^, ^48^ some of the study participants that reported functionally normal hearing when using hearing aids might have had limited awareness of what functionally normal hearing is by the time they began to use hearing aids.

Although ADRC staff were trained and instructed to report on whether they felt participants’ ability to answer the CDR‐SB questions was impacted by their hearing loss, there is a risk that hearing loss may have confounded cognition results for some participants. Moreover, even though standard criteria and procedures were applied across all ADRCs, there may be some differences in diagnostic definitions and variability in recruitment strategies implemented by each ADRC.

Finally, education level and income of ADRC cohort participants are likely higher than the national average and they are predominantly White. Participants who chose to use hearing aids may have differed from those who did not by factors including higher socioeconomic status and education. Therefore, compared to an RCT in which exposure is randomly assigned, the characteristics of participants in our study were unbalanced between exposed and unexposed groups. To address the bias, due to non‐randomized exposure allocation, potential confounding effects of both time‐varying and baseline covariates and selection bias due to drop out, we implemented the IPW approach to fit the MSC model. The IP weighting allowed us to assign exposure/treatment independently of the counterfactual responses and conditional on observed covariates and calculate statistics standardized to a pseudo‐population in which the exposure was independent of the measured confounders. Although the implementation of the IPW‐adjusted MSC models accounted for a large set of measured covariates, selected based on evidence from previous work, there is still a chance that our study may include unmeasured confounding factors that were unintentionally omitted from the analysis. These unmeasured confounders could possibly bias causal inferences in our analysis. To account for the impact of potential unmeasured confounding bias on the associations, we performed the sensitivity analysis for unmeasured confounding. Given that our conclusions remained robust over a wide range of plausible assumptions, thus reducing the number of interpretations of our findings, the causal nature of the observed associations is more defensible.

More research is needed to better understand the relationship between hearing impairment and changes in cognitive ability and the role of hearing rehabilitative strategies in mitigating these effects. While waiting for further studies with more objective measurement of audiological deficits facts on the audiological side to complement the cognitive data, the present study provides an important support for links among hearing impairment, hearing aid use, and progression to MCI in cognitively healthy adults. Although causality remains to be determined, we carefully infer that increased access to quality hearing health care might prove an effective preventive intervention to mitigate the impending dementia epidemic.

## CONFLICTS OF INTEREST

All authors declare that they have no competing interests.

## AUTHOR CONTRIBUTIONS

Magda Bucholc, Paula L. McClean, Sarah Bauermeister, Stephen Todd contributed to study design. Magda Bucholc did the data analysis and data interpretation. Magda Bucholc drafted the manuscript. Sarah Bauermeister, Daman Kaur, Paula L. McClean, and Stephen Todd critically reviewed the manuscript. All authors had full access to all the data in the study and had final responsibility for the decision to submit for publication.

## Data Availability

Data can be obtained through the National Alzheimer's Coordinating Center data request process (https://naccdata.org/requesting‐data/data‐request‐process).
